# Supplementary material for: Positive Effect of Lactobacillus acidophilus EG004 on Cognitive Ability of Healthy Mice by Fecal Microbiome Analysis Using Full-Length 16S-23S rRNA Metagenome Sequencing
Source: Microbiol Spectr. 2022 Jan 12;10(1):e01815-21. doi: 10.1128/spectrum.01815-21 (PMC8754107; doi:10.1128/spectrum.01815-21)
Supplement: SUPPLEMENTAL FILE 1 — Supplemental material. Download SPECTRUM01815-21_Supp_1_seq10.pdf, PDF file, 0.9 MB [file spectrum01815-21_supp_1_seq10.pdf]

Supplementary Information

Contents

Supplementary Figures

Figure S1. Phylogenetic tree using 16S rRNA of three probiotic strains 2

Figure S2. Animal body weight changes by week 3

Figure S3. Comparison of microbial composition between the group fed *L. acidophilus* and control ... 4

Figure S4. Circularized genome of *L. acidophilus* EG004 5

Figure S5. Circularized genome of *Lcb. paracasei* EG005 6

Figure S6. Circularized genome of *Lcb. rhamnosus* EG006 7

Supplementary Tables

Table S1. Results of cognitive behavioral tests 8

Table S2. SCFA identification in bacterial culture 9

Table S3. Cognitive ability assessment score 10

Supplementary Figures

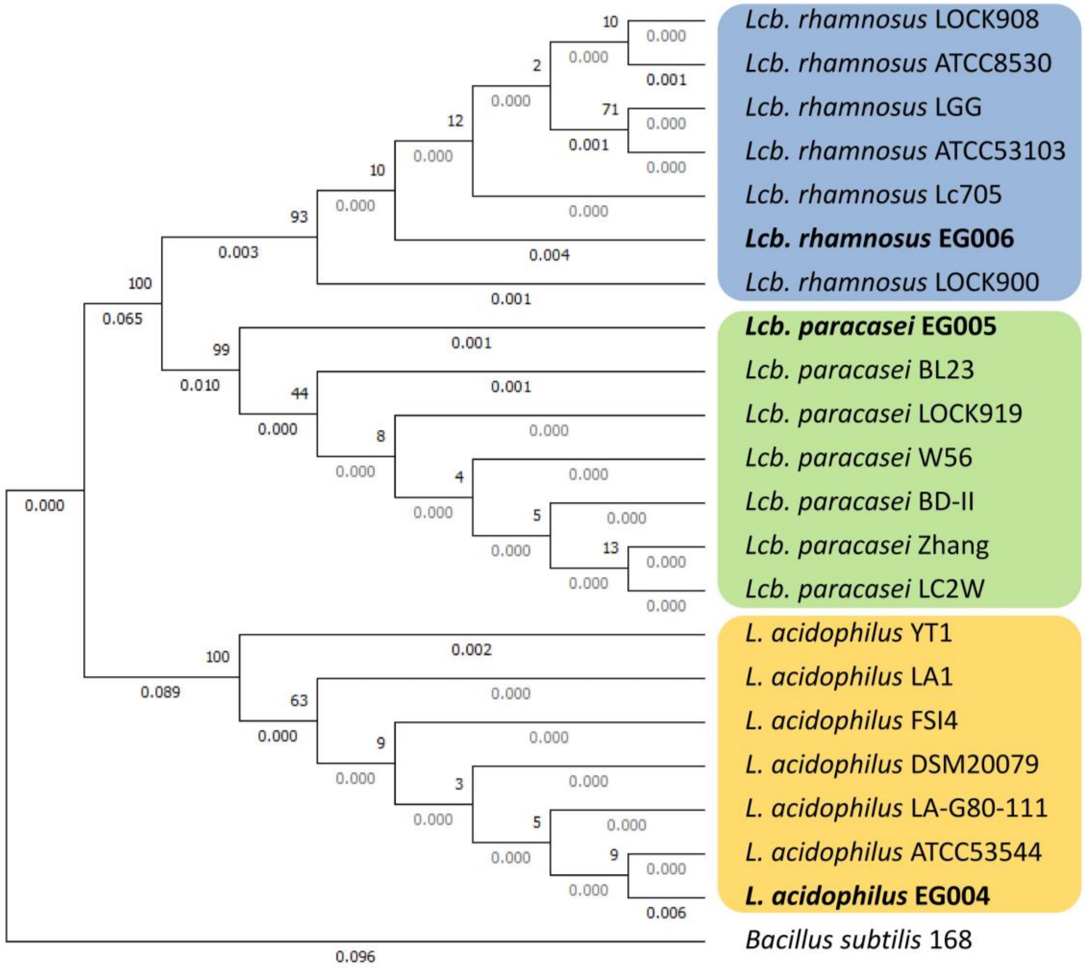

Figure S1. Phylogenetic tree using 16S rRNA of **three probiotic strains**

Phylogenetic tree by Maximum likelihood method based on 16S rRNA sequences for 14 *Lacticaseibacillus* genera and 7 *L. acidophilus* species including three strains in our study. Bootstrapping was conducted 1000 times, and the *Bacillus subtilis* 168 was used as an outgroup. The same species were enclosed in colored boxes, and three strains used in our study were presented in bold font.

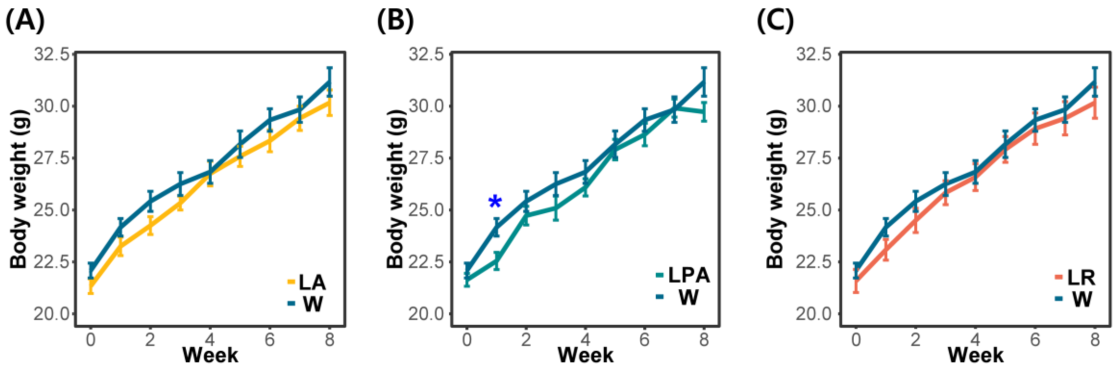

Figure S2. Animal body weight changes by week

We measured body weight of the mice every week. The average difference was found between the group fed *Lcb. paracasei* and control, but it was immediately recovered.

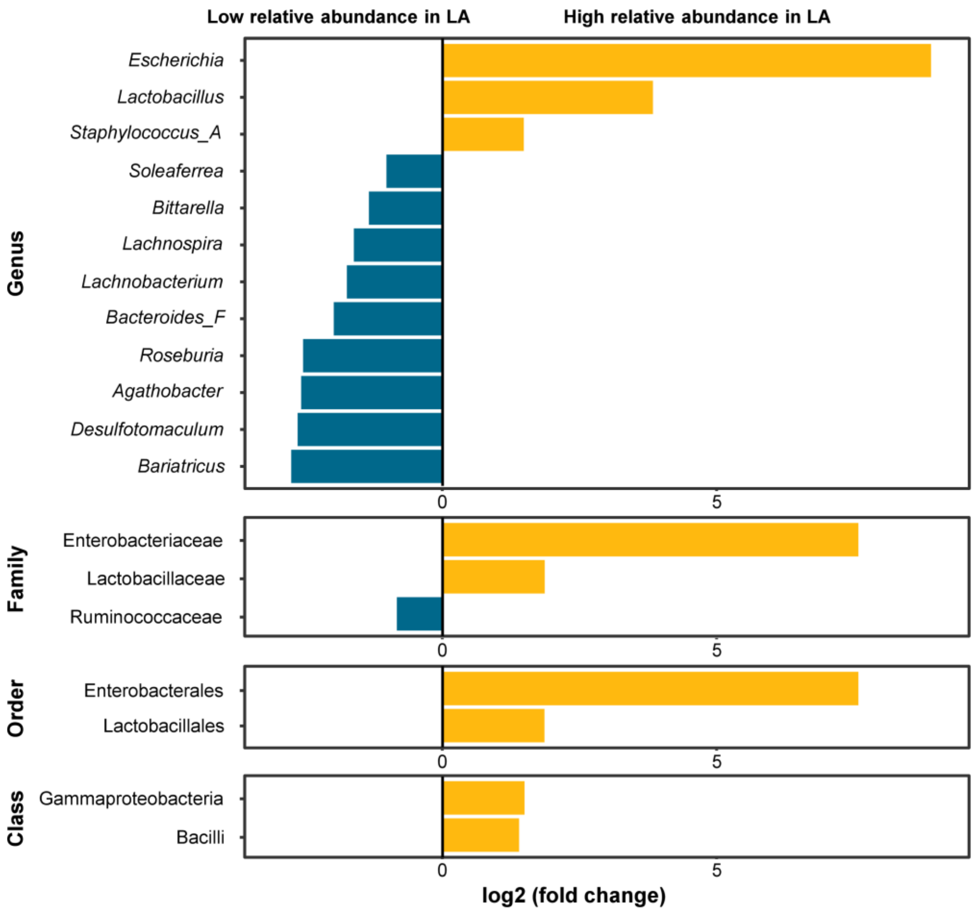

Figure S3. Comparison of microbial composition between the group fed *L. acidophilus* and control

We compared microbial composition of the group fed *L. acidophilus* and control at all taxonomic levels. The taxonomy found with high relative abundance in the group fed *L. acidophilus* showed yellow bar, while the other showed turquoise colored bar.

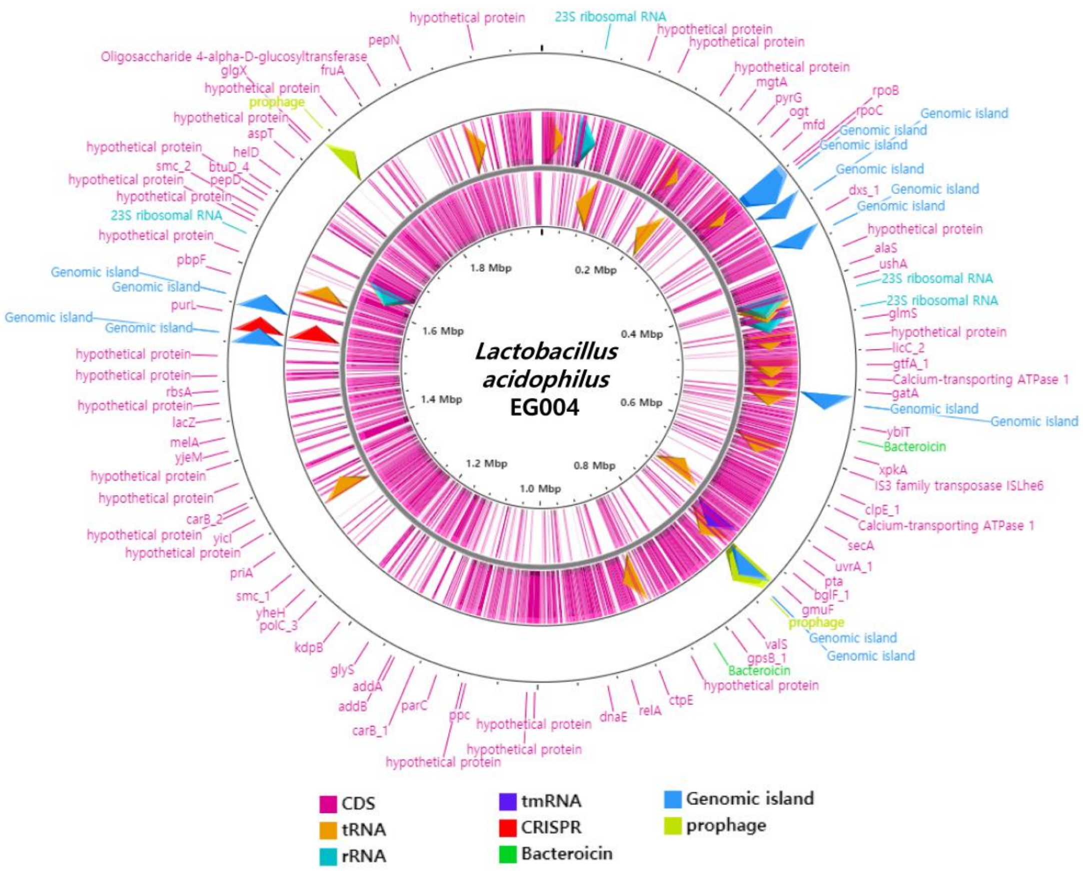

Figure S4. Circularized genome of *L. acidophilus* EG004

The genetic map represents main genetic factors of *L. acidophilus* EG004. There are three rings in generated, which indicates potential virulence factors (outermost ring), genes encoded on the forward strand, genes encoded on the backward stand (innermost ring).

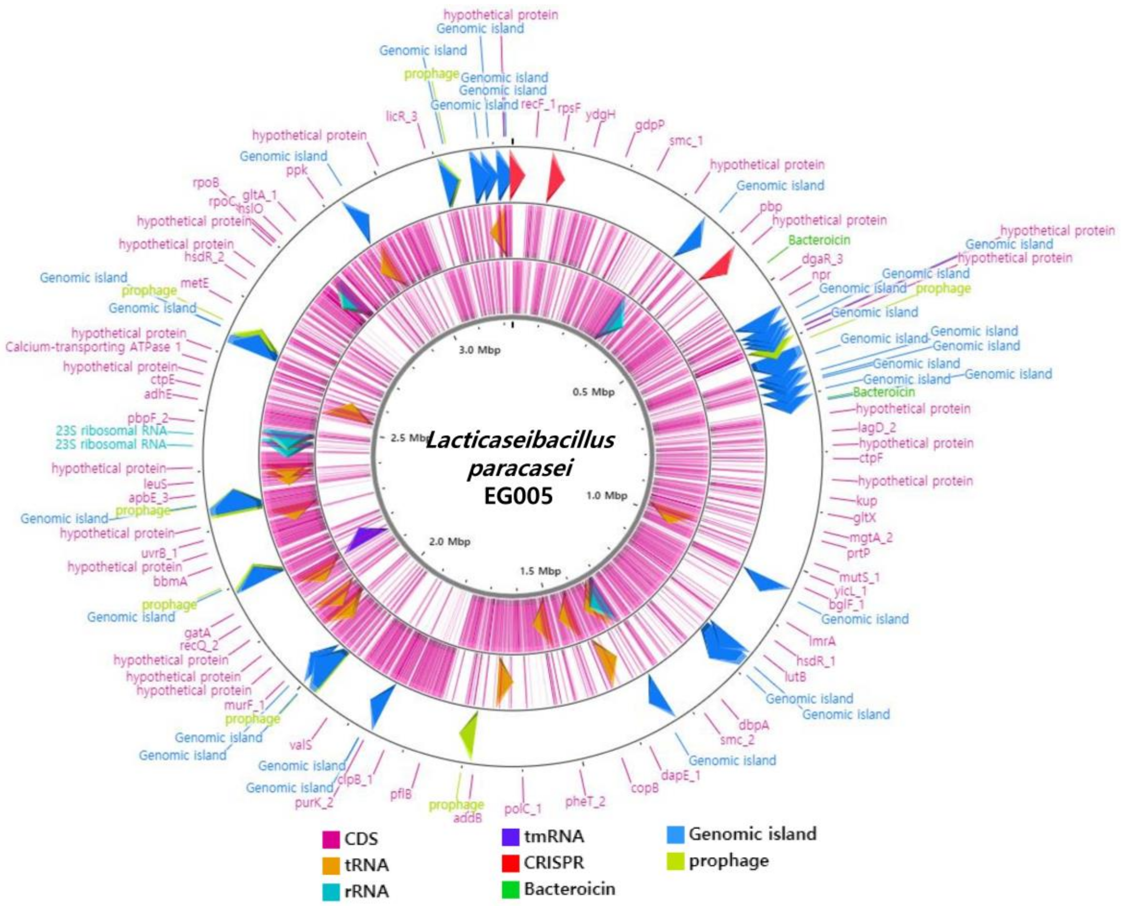

Figure S5. Circularized genome of *Lcb. paracasei* EG005

This genetic map represents main genetic factors of *Lcb. paracasei* EG005. There are three rings in generated, which indicates potential virulence factors (outermost ring), genes encoded on the forward strand, genes encoded on the backward stand (innermost ring).

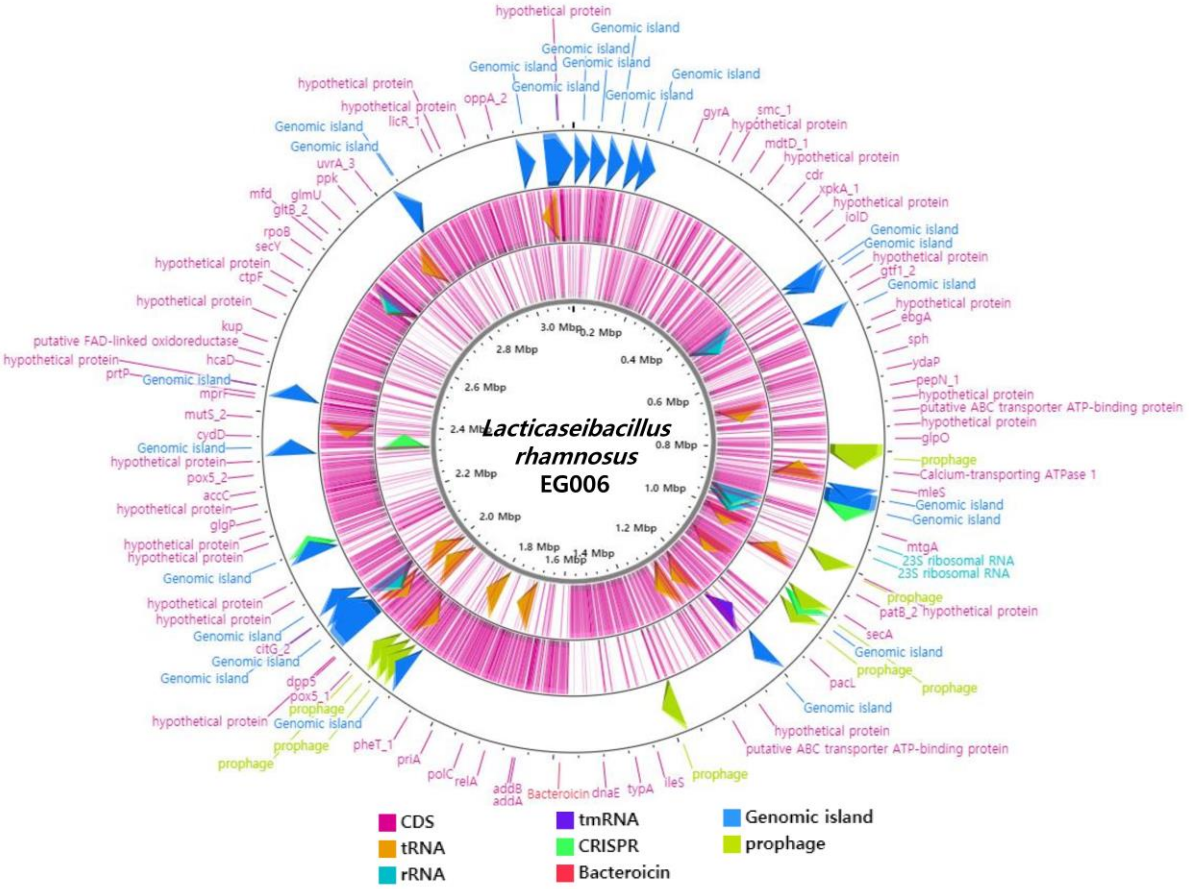

Figure S6. Circularized genome of *Lcb. rhamnosus* EG006

The physical map shows main genetic factors of *Lcb. rhamnosus* EG006. There are three rings in generated, which indicates potential virulence factors (outermost ring), genes encoded on the forward strand, genes encoded on the backward stand (innermost ring).

Supplementary Tables

Table S1. Results of cognitive behavioral tests

Test	Assessment parameter	LA[a]	LPA[b]	LR[c]	Control
SA	Spontaneous alternation [*][%]	*66.08±6.11*	62.36±4.96	59.42±4.51	52.02±2.58
	Total arm entries	13.83±0.41	11.36±0.34	13.75±0.27	13.83±0.36
	Group ratio [d,*] [%]	*75.00*	63.64	58.33	66.67
NOR	Discrimination ratio [*] [%]	63.39±4.59	*64.53±6.06*	51.50±2.40	46.92±4.89
	Group ratio [e, *] [%]	*83.33*	72.73	41.67	60.00
	Time $_{Familiar}$ [f] (s)	4.10±0.76	2.75±0.68	4.83±0.92	4.32±0.81
	Time $_{Novel}$ [g] (s)	6.73±0.92	5.74±1.29	5.47±1.30	3.68±0.61
	Number $_{Familiar}$ [h]	5.17±0.65	3.00±0.66	5.92±0.72	5.50±0.95
	Number $_{Novel}$ [i]	7.75±0.91	5.45±0.92	7.50±1.59	5.40±0.83
PAT	Step latency at day 1 (s)	43.62±8.54	21.85±5.38	67.69±27.31	23.60±6.52
	Step latency at day 2 [*] (s)	*300±0.00*	281.20±14.05	292.59±7.41	193.79±41.97
FA	Forced alternation [*] [%]	28.92±5.72	20.63±5.04	*37.90±3.84*	23.33±6.03
	Total arm entries	18.20±3.09	10.71±0.81	17.36±1.16	15.42±1.73
	Group ratio [j, *] [%]	*83.33*	55.56	66.67	66.67

[a]: the group fed *L. acidophilus*, [b]: the group fed *Lcb. paracasei* [c]: the group fed *Lcb. rhamnosus*, [d]: The ratio of the mouse entered spontaneous alternation at first 3 entries, [e]: ratio of the mouse touched novel object at first, [f~i]: Time and number of touched Familiar or Novel objects, [j]: ratio of the mouse entered novel arm, and [*]: items for cognitive ability evaluation score. The highest value of cognitive ability indicators among the experimental groups was represented with red colored italic font. All values are shown as the mean ± SEM.

Table S2. SCFA identification in bacterial culture

Strain	Ret.Time (min)	Peak Name	Height (μRIU)	Area (μRIU*min)	Rel.Area (%)	Amount (mg/L)	Rel.Amount (%)
EG004	15.66	Lactic acid	72.540	27.036	81.58	13493.703	74.55
	18.69	Acetic acid	14.314	6.103	18.42	4605.749	25.45
	Total		86.855	33.139	100.00	18099.452	100.00
EG005	15.65	Lactic acid	80.019	29.558	83.96	14752.368	77.58
	18.68	Acetic acid	13.566	5.649	16.04	4262.900	22.42
	Total		93.585	35.207	100.00	19015.268	100.00
EG006	15.65	Lactic acid	50.834	18.616	77.89	9291.048	69.96
	18.68	Acetic acid	12.576	5.286	22.11	3989.009	30.04
	Total		63.410	23.901	100.00	13280.058	100.00

Short-chain fatty acids were measured by HPLC analysis. Broth media cultured for 24 hours were used for the analysis.

Table S3. Cognitive ability assessment score

Test	Evaluation item	LA[a]	LPA[b]	LR[c]	Control
SA	Spontaneous alternation [%]	4	3	2	1
	Group ratio [d] [%]	4	2	1	3
NOR	Discrimination ratio [%]	3	4	2	1
	Group ratio [e] [%]	4	3	1	2
PAT	Step latency at day 2 (s)	4	2	3	1
	Forced alternation [%]	3	1	4	2
FA	Group ratio [i] [%]	4	1	2	2
	Total	26	16	15	12

[a]: the group fed *L. acidophilus*, [b]: the group fed *Lcb. paracasei* [c]: the group fed *Lcb. rhamnosus*, [d]: The ratio of the mouse entered spontaneous alternation at first 3 entries, [e]: ratio of the mouse touched novel object at first, and [i]: ratio of the mouse entered novel arm. Scores of each cognitive ability assessment were given in ascending order of ranking (1-4 points).
